# Editorial: Immune cell therapy approaches targeting tumor microenvironment

**DOI:** 10.3389/fimmu.2025.1628689

**Published:** 2025-05-28

**Authors:** Santosh K. Patnaik, Suresh Gopi Kalathil

**Affiliations:** ^1^ Department of Thoracic Surgery, Roswell Park Comprehensive Cancer Center, Buffalo, NY, United States; ^2^ Department of Surgery, Jacobs School of Medicine and Biomedical Sciences, State University of New York, Buffalo, NY, United States

**Keywords:** cancer, combination therapy, immunotherapy, tumor microenvironment, NK cells, immune checkpoint

## Introduction

Recent advances in immunotherapy have significantly improved clinical outcomes, particularly for cancers that were once considered untreatable or with poor prognosis. A growing body of evidence underscores the importance of both tumoral and non-tumoral components of the tumor microenvironment (TME) in driving cancer progression. The TME is a dynamic and complex ecosystem that includes not only cancer cells but also stromal cells such as immune cells, endothelial cells, and fibroblasts, as well as extracellular matrix components. These constituents can act jointly to create a pro-tumorigenic environment that facilitates tumor growth, metastasis, and therapeutic resistance, in particular, through immunosuppression, metabolic reprogramming, and angiogenesis. Interactions between cancer and other cells of the TME can also promote cancer cell heterogeneity, clonal evolution, and development of drug resistance mechanisms. Because of these reasons, the TME holds significant promise for improving cancer therapies. The genetic stability of the TME also makes it a compelling target for therapeutic interventions.

The articles in this Research Topic provide valuable insights that inform the development of innovative therapeutic strategies to target the TME in order to pave the way for improvised approaches for cell-based cancer immunotherapy. The findings of Puebla-Osorio et al. underscore the therapeutic potential of combining chimeric antigen receptor (CAR) T cell therapy with low-dose radiotherapy, particularly when tailored to specific tumor types and when the adoptively transferred effector T cells are precisely matched with radiation dosage. This innovative strategy has demonstrated promise across a range of preclinical models and holds significant potential for translation into clinical trials. Notably, it offers the unique advantage of eliciting therapeutic effects without incurring radiation-induced tissue damage. A potential role of ITGAL integrin in the immune landscape of non-small cell lung cancer is described by Zhang et al. Downregulation of ITGAL in tumors of this cancer as well as its association with poor prognosis indicate that ITGAL can be a valuable biomarker for predicting outcomes. The correlation between ITGAL expression and tumor infiltration by CD8^+^ T cells and macrophages implies that modulation of ITGAL expression or function can be a strategy to enhance anti-tumor immunity. The microbiome study of patients with acute myeloid leukemia by Zhong et al. underscores the growing recognition of the influence of intestinal microbiota on cancer. The study illustrates that antibiotics, besides controlling infections, can inadvertently affect the prognosis by altering key aspects of the immune biology and chemotherapy sensitivity. The work of Agostini et al. demonstrates that a nutritional medium rich in specific growth factors can enhance the migration of adipocyte stem cells. Such media may have therapeutic potential for cancer as well as regenerative applications.

Besides the above-mentioned research studies, the Research Topic includes a few reviews that bring us up to date on critical aspects of TME regarding immunotherapy. Li et al. review our understanding of homing of immune, stem, and circulating cancer cells to tumors. This work suggests that refining the homing ability of cells, especially in combination with other therapies, could be a strategy for cancer treatment. Chen et al. provide a comprehensive overview of the distinct cellular components of the ovarian cancer TME, elucidating their roles and associated pathways. Neutrophil extracellular traps (NETs), which are formed from DNA released by activated neutrophils, can impact cancer within the TME. Chen et al. provide a comprehensive overview of the latest research findings on the interaction between NETs and cancer, particularly in gynecologic tumors, serving as a valuable resource for future exploration in this field. The work of Qi et al. examines TME modulation by agonists of STING (stimulator of interferon genes) as a promising strategy to enhance immunotherapy efficacy. By targeting the immunosuppressive components of the TME, this approach aims to convert immunologically “cold” tumors into “hot” ones rendering them more susceptible to immune-mediated attack. Xu et al. highlight the intricate balance between different interleukins in the TME that influence tumor surveillance by NK cells. Targeting these cytokine pathways presents a promising strategy for enhancing NK cell-mediated tumor clearance and improving breast cancer outcomes. Engineering cytokines like IL-15, IL-12, and IL-18, along with the development of bispecific and trispecific antibodies, are likely to play a significant role in improving NK cell function within the TME. The immunosuppressive nature of TME in liver cancer remains a significant barrier to effective treatment. This immunosuppression arises from immune checkpoint molecules such as programmed death-1 (PD-1) and immunosuppressive cytokines that cause T cell exhaustion and inhibition. Immunotherapy, particularly with immune checkpoint inhibitors (ICIs), has shown great potential in improving outcomes for patients with advanced or unresectable hepatocellular carcinoma (HCC). The review by Hao et al. summarizes the latest clinical advancements in treatment strategies combining immunotherapy with targeted therapies for HCC treatment and discusses future directions and challenges in optimizing these regimens. Lastly, Dong et al. review current literature on the clinical benefit of combining ICIs with JAK inhibitors for enhancing anti-tumor immunity and overcoming resistance to immunotherapy in lung cancer and Hodgkin lymphoma.

